# NISC: Neural Network-Imputation for Single-Cell RNA Sequencing and Cell Type Clustering

**DOI:** 10.3389/fgene.2022.847112

**Published:** 2022-05-03

**Authors:** Xiang Zhang, Zhuo Chen, Rahul Bhadani, Siyang Cao, Meng Lu, Nicholas Lytal, Yin Chen, Lingling An

**Affiliations:** ^1^ Interdisciplinary Program in Statistics and Data Science, University of Arizona, Tucson, AZ, United States; ^2^ Department of Biosystems Engineering, University of Arizona, Tucson, AZ, United States; ^3^ Department of Electrical and Computer Engineering, University of Arizona, Tucson, AZ, United States; ^4^ Department of Mathematics and Statistics, California State University at Chico, Chico, CA, United States; ^5^ College of Pharmacy, University of Arizona, Tucson, AZ, United States; ^6^ Department of Biostatistics and Epidemiology, University of Arizona, Tucson, AZ, United States

**Keywords:** imputation, deep learning, single cell RNA-seq, dropout, autoencoder

## Abstract

Single-cell RNA sequencing (scRNA-seq) reveals the transcriptome diversity in heterogeneous cell populations as it allows researchers to study gene expression at single-cell resolution. The latest advances in scRNA-seq technology have made it possible to profile tens of thousands of individual cells simultaneously. However, the technology also increases the number of missing values, i. e, dropouts, from technical constraints, such as amplification failure during the reverse transcription step. The resulting sparsity of scRNA-seq count data can be very high, with greater than 90% of data entries being zeros, which becomes an obstacle for clustering cell types. Current imputation methods are not robust in the case of high sparsity. In this study, we develop a Neural Network-based Imputation for scRNA-seq count data, NISC. It uses autoencoder, coupled with a weighted loss function and regularization, to correct the dropouts in scRNA-seq count data. A systematic evaluation shows that NISC is an effective imputation approach for handling sparse scRNA-seq count data, and its performance surpasses existing imputation methods in cell type identification.

## 1 Introduction

Single-cell RNA sequencing (scRNA-seq) is designed to profile gene expression at the single-cell level, making it possible to study the heterogeneity among individual cells (Pierson and Yau, 2015). However, one important characteristic of scRNA-seq data is a phenomenon called “dropout”, which causes challenges in data analysis. These dropout events occur because of the low amounts of genetic material in individual cells and inefficient mRNA capture, as well as the stochasticity of mRNA expression (Lin et al., 2017). Specifically, a large number of dropouts is due to transcripts lost in the RNA reverse transcription procedure during library preparation (Gordon et al., 2015). In other words, many zero counts in the gene expression data are not “true” values. Consequently, the scRNA-seq data may be incredibly sparse due to the high dropout rate, e.g., more than 90% of the expression counts have values of zero. Imputation has become an essential preprocessing step for downstream analysis of scRNA-seq data (Tracy et al., 2019). Recent studies have shown that some imputation methods improve downstream analysis and have already been implemented in scRNA-seq analysis pipelines (Zhang and Zhang, 2018). Meanwhile, with the increasing size of scRNA-seq data sets, appropriate imputation methods are necessary to compensate for these dropouts to reduce the impacts of missing values (Angerer et al., 2017).

Many methods have recently been developed for modeling and processing scRNA-seq count data, including scVI (Lopez et al., 2018), VASC (Wang and Gu, 2018), scSVA (Sun et al., 2019), scVAE (Gronbech et al., 2020), and scAEspy (Tangherloni et al., 2021), which used neural networks to reduce the noisy dimension to increase the accuracy of downstream analysis. There also exists quite a number of methods to impute the missing values in scRNA-seq data, including scImpute, MAGIC (Van Dijk et al., 2018), SAVER (Huang et al., 2018), DrImpute (Gong et al., 2018), VIPER (Chen and Zhou, 2018), ALRA (Linderman et al., 2018), EnImpute (Zhang et al., 2019) and scDoc (Ran et al., 2020). In ScImpute, separated Gamma-Normal mixture models are constructed for different cell subgroups to calculate the probabilities of drop-out. It leverages information of cell similarity in terms of genes with a lower dropout probability and then imputes the values of genes with higher dropout probability. MAGIC is a method that shares information across similar cells *via* data diffusion to predict the true gene expression level. SAVER is a Bayesian-based imputation method that imputes dropout values and generates a substitution for each gene. DrImpute is a clustering-based method that generates estimations using cluster priors and distance matrices. ALRA is an adaptively-thresholded low-rank approximation method that rescales the scRNA-seq expression matrix using randomized singular value decomposition. VIPER is a statistical method that fits a linear model for each cell by cell-cell interaction.

Basically, these methods impute dropouts by leveraging information on similarities between cells/genes using the correlation structure of the scRNA-seq data. For example, current imputation approaches, including scImpute and DrImpute, identify similar cells/genes based on clustering and then impute the missing data by averaging the gene expression values for each detected cluster. The accuracy of these imputation methods highly relies on clustering analysis. EnImpute combines the imputation results obtained from eight different imputation methods and calculates the expected values. scDoc imputes dropout events by leveraging information for the same gene from highly similar cells. However, current methods may fail to capture the nonlinearity and the count structure of the scRNA-seq data. Moreover, it becomes more challenging for the traditional imputation methods to handle datasets with increasing size (Eraslan et al., 2019).

Recently, some deep learning-based imputation methods have been developed for efficiently handling the higher dimensional scRNA-seq data, such as DCA (Eraslan et al., 2019), DeepImpute (Arisdakessian et al., 2019), AutoImpute (Talwar et al., 2018), LATE (Badsha et al., 2020), scIGAN (Xu et al., 2020), and scGNN (Wang et al., 2021). DCA is a neural network-based denoising method for scRNA-seq count data. This method assumes that the scRNA-seq count data follow a negative binomial distribution and then are denoised by maximizing a likelihood function. DeepImpute is a deep learning-based method that splits the genes into several subsets of neural networks. However, these imputation methods lack accuracy and power in handling highly sparse data. AutoImpute uses autoencoder with one hidden layer to impute missing values in scRNA-seq data by minimizing the Euclidean cost function. LATE uses autoencoder to train on nonzero data by minimizing the loss function, therefore imputing the missing values based on information of dependence between genes and cells. scIGAN uses generative adversarial networks for scRNA-seq imputation. scGNN uses a graph neural network for scRNA-seq analysis.

In this study, we develop a novel imputation method, Neural Network-based Imputation for scRNA-seq data (NISC) to improve cell type clustering. It is based on neural networks with a novel weighted loss function, coupled with regularizations. Through a series of simulation studies and real data analysis, NISC is compared with the other imputation methods, including AutoImpute, DCA, DeepImpute, LATE, SAVER, MAGIC, ScImpute, DrImpute, EnImpute, ALRA, VIPER, scDoc, scIGAN, and scGNN. The results show that NISC outperforms the existing imputation methods as it can recover the gene expression more correctly and distinguish the cell types more precisely, particularly for scRNA-seq data with high sparsity/noise.

## 2 Methods

### 2.1 Neural Network Architecture

**FIGURE 1:**
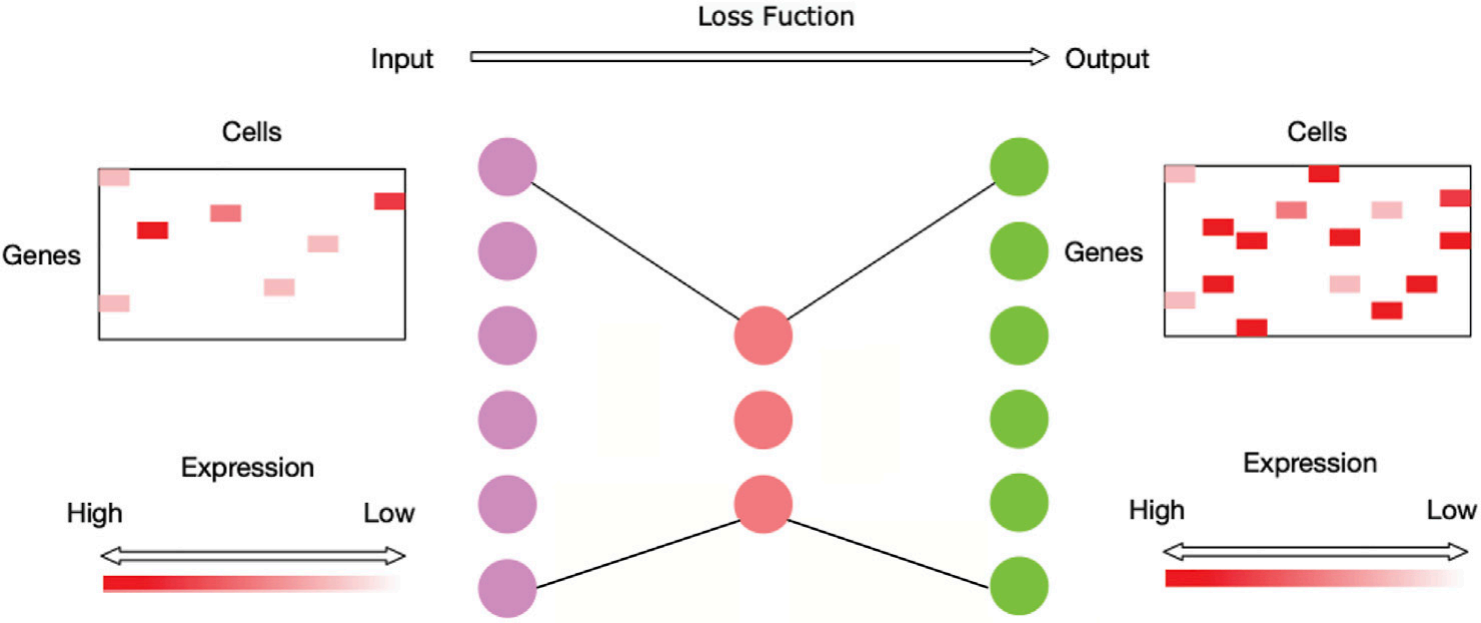
Neural network architecture of NISC. This network is mainly composed of three hidden layers (i.e., dots with three different colors, purple, red, and green). The first hidden layer has neurons equal to twice the number of genes of the input data. It is followed by the second hidden layer in the middle with neurons equal to around half the number of genes of the input data. The third layer has neurons equal to the number of neurons of the first layer. This neural network is trained using an optimization process with a loss function to calculate the model error.

It is evident that the process of imputing the dropouts for scRNA-seq data is similar to the process of outlining a noisy image, so autoencoder is utilized to impute the sparse scRNA-seq data (Shao et al., 2013). Autoencoder is an unsupervised learning technique that has been used in image denoising (Vincent et al., 2010). The autoencoder technique allows nonlinear data vectors to be stacked, making the technique more powerful and able to learn complicated relations between layers (Mao et al., 2016). An autoencoder model consists of an encoder and a decoder. An encoder stage compresses the input data into a low-dimensional code, and then a similar decoder stage reconstructs the output data from the code (Hinton and Salakhutdinov, 2006). Figure 1 shows the neural network architecture of NISC. The number of neurons for the hidden layer in the middle is usually much smaller than the number of neurons for the input/output layers to reduce the redundant information in data. In our method NISC, the number of neurons in the neural network architecture is set to be proportional to the number of genes.

### 2.2 Loss Function and Regularizations

It has been found that the main reason for dropouts in scRNA-seq data is due to failure of the reverse transcription of mRNA (Bengtsson et al., 2005; Reiter et al., 2011). Reverse transcription is an enzyme reaction; therefore, the Michaelis-Menten function can be used to model the relationship between dropout probability and gene expression for full-transcripts scRNA-seq data (Andrews and Hemberg, 2019). The following equation shows the dropout probability *P*_*ij*_ for the gene *i* in cell *j* using Michaelis-Menten kinetics (MMK) (Brennecke et al., 2013),

$$P_{ij}=1-\frac{S_{ij}}{K_{M}+S_{ij}}$$ (1)

where *S*_*ij*_ is the observed gene expression level of gene *i* in cell *j*, and *K*_*M*_ is the Michaelis constant (Johnson and Goody, 2011). We use this probability to describe the dropout event, which will then be involved in the calculating the network’s denoised output.

We propose a novel loss function with the mean square error weighted by the dropout probability estimated through Michaelis-Menten kinetics.

$$Loss=\sum_{i=1}^{m}\sum_{j=1}^{n}\left(1-P_{ij}\right)\cdot {\left(\text{log}\left({\hat{y}}_{ij}\right)-\text{log}\left(y_{ij}\right)\right)}^{2}+\alpha \cdot {\left\|\beta \right\|}_{2}$$ (2)

The loss function will be minimized through the autoencoder learning process. Note: the function “log” is the natural logarithm. The intuition behind this is that the estimated dropout probability $P_{ij}$ affects the loss function adversely. In this manner, the imputed gene expression ${\hat{y}}_{ij}$ will be close to the observed gene expression $y_{ij}$ when the estimated dropout probability $P_{ij}$ is low. When we train an autoencoder network, a challenging problem is how to avoid overfitting. Overfitting refers to a neural network model that fits the training data too well to predict the pattern of new data. Overfitting is caused by noise in the training data, and the neural network includes this noise during the learning process. To avoid overfitting, we need to reduce the complexity of the network; therefore, we applied *L*_*2*_ regularization (ridge regression) and dropout regularization to reduce the complexity of the autoencoder network (note: this is different from the term “dropout” event in scRNA-seq data). It is the first time that these two regularization techniques have been combined with an autoencoder network for imputation of scRNA-seq data. We define the regularization term ${\left\|\beta \right\|}_{2}$ as the *L*_*2*_ norm of the weight matrix, that is, the sum of all squared weight values of the matrix (i.e., the first term in the above loss function). *α* is defined as the value of the regularization rate, which determines how powerful the effect of the regularization term will be. The regularization term ${\left\|\beta \right\|}_{2}$ is weighted by the scalar *α* and the regularization term will be excluded if *α* is zero. If *α* is too large, the neural network model will be less sensitive therefore increase the risk of underfitting. Conversely, if *α* is too small, the complexity of the model will be increased, so the risk of overfitting will be high. An appropriate value of *α* can be determined through cross-validation suggested by Ng et al.(2004).

In addition to *L*_*2*_ regularization, dropout regularization is also used in NISC as it is a strategy to turn off neurons of the neural network with certain probability during training, which then further reduces the model’s complexity (Srivastava et al., 2014). Furthermore, to mitigate the effect of reaching the local optimization peak by the neural network, the Adaptive Moment estimation algorithm is used to perform stochastic optimization (Eweda and Macchi, 1984).

### 2.3 Performance Evaluation

The proposed method is compared with the existing imputation methods through a series of simulated datasets and three real datasets. First, we visualize cell type sub-populations using 2-dimensional PCA (principal component analysis) plots or t-SNE (t-distributed stochastic neighbor embedding) plots (Kin et al., 2002; Kobak and Berens, 2019) depending on the data property (Anowar et al., 2021). UMAP (uniform manifold approximation) plots are also drawn (Becht et al., 2019). The commonly used unsupervised clustering algorithms, k-means (Na et al., 2010) and hierarchical clustering algorithms (Murtagh and Contreras, 2017), and Leiden algorithm(Traag et al., 2019), are used to group the cells on the reduced dimension of visualization results, which can then be used for calculating the performance measurements of each imputation method.

Four evaluation metrics are calculated to evaluate the accuracy of the cell type clusters in the visualization plots, including Adjusted Mutual Information (AMI) (Romano et al., 2014), Adjusted Rand Index (ARI) (Steinley, 2004), Fowlkes-Mallows Index (FMI) (Nemec and Brinkhurst, 1988), and Silhouette Score (SS) (Rousseeuw, 1987). Since we know the truth for the simulated data, the RMSE (Root Mean Square Error) is also calculated between the imputed values and the truth to assess the performance of imputation methods (Blondel et al., 2008; Skinnider et al., 2019). Additionally, the heatmap of gene expression in the simulated studies is also drawn to demonstrate the direct comparison of the methods in detail.

## 3 Results

### 3.1 NISC Enhances Cell Type Visualization in Simulated scRNA-Seq Data

To evaluate the performance of our imputation method, we compare it with existing methods on simulated scRNA-seq count data, which are generated by the widely used simulator, Splatter (Zappia et al., 2017). Both raw count data with dropouts/noise and its corresponding true data are available through simulations. The raw count data is the input data of the learning framework, and the ground truth data can be used to assess the performance of imputation. The count data are represented as an expression matrix, where each row is a gene, and each column is a cell. We consider three scenarios:

(1) Two cell types for 800 genes and 1,000 cells.
(2) Four cell types for 800 genes and 1,000 cells
(3) Four cell types for 2,000 genes and 10,000 cells

For each scenario, two sparsity levels are examined, i.e., approximately 80 vs 90%. In the Splatter simulation setting, the differential rate of 0.2 is used, indicating that 20% of the total genes are marker genes. As substantial noise is added to input data to mask cell type identities through simulation, our purpose is to predict the imputed values for the dropouts accurately and therefore identify cell types.

Our deep learning framework in NISC consists of three hidden layers with 1600, 400, and 1600 neurons, respectively, for the simulation data of 800 genes. For the case of 2,000 genes, the number of neurons for three hidden layers are 4,000, 1,000, and 4,000, respectively. A widely used active function, rectified linear unit (Xing et al., 2016), is employed to train each cell to capture the nonlinearity of the data. The number of neurons for the encoder/decoder layers is twice the number of genes, while the number of neurons for the hidden layer in the middle of the architecture is half of the number of genes. We compare NISC to other existing imputation methods in simulation data for various scenarios. The figures below are for the scenario (2). Some representative results for scenario 1) and 3) are included in the Supplementary File.

**FIGURE 2:**
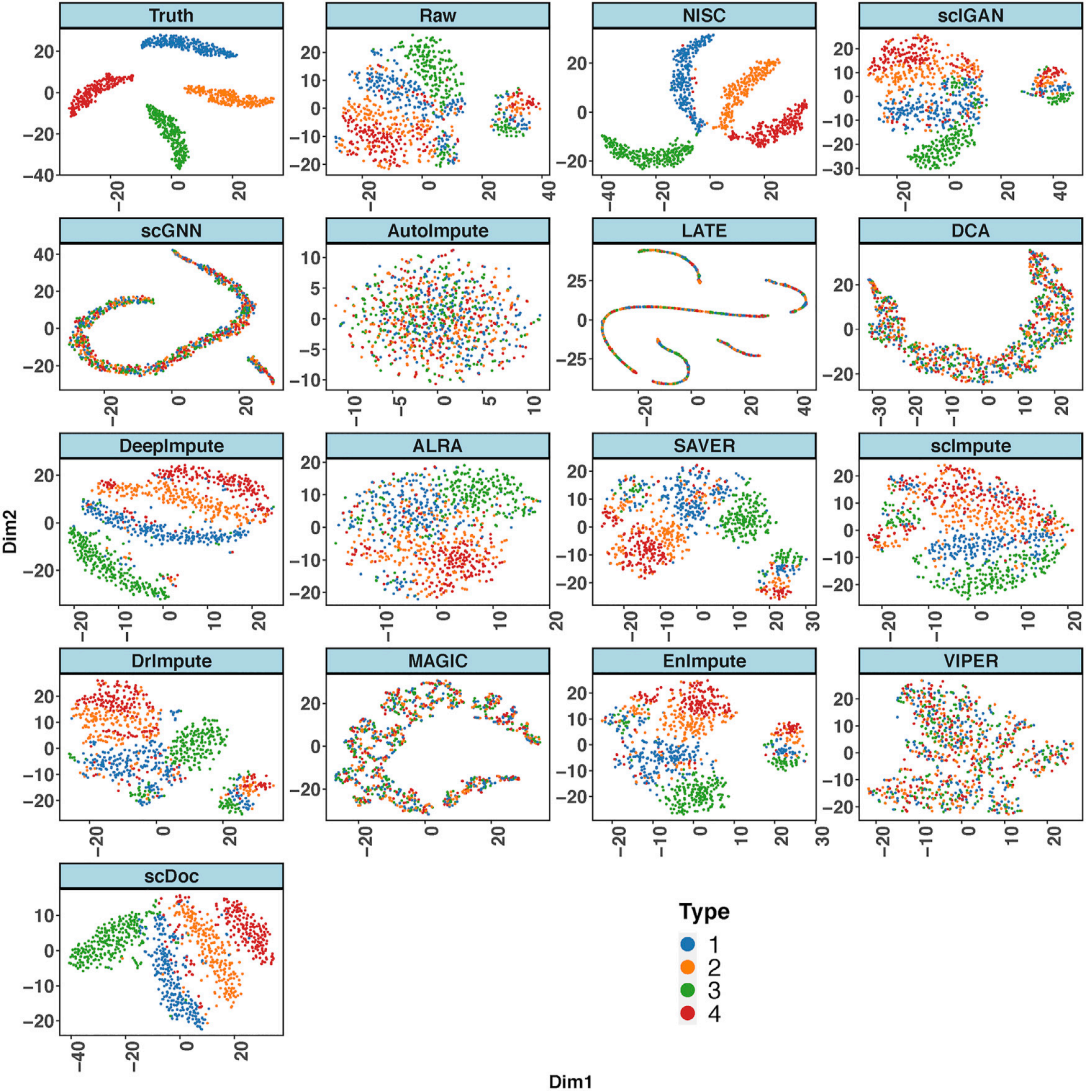
NISC significantly improves the performance of t-SNE in visualizing simulated scRNA-seq count data. Plots of the first two components are calculated from the simulated ground truth data, raw data, and imputed data using various imputation methods. The dataset contains 800 genes and 1,000 cells in 4 cell types, with 90% sparsity. Cells are colored by cell types as indicated.

Figure 2 shows the t-SNE plots derived from the ground truth of cells, the raw input data, and the imputed data by NISC and other existing methods. The ground truth contains 4 cell types while the types are mixed in the raw data. This is due to the high sparsity (i.e., high noise, 90% data are zeros) in the raw input, which distorts the topology of the ground truth. NISC can accurately recover the dropouts, and the cells are clearly located in four groups/clusters, followed by scDoc and DeepImpute. However, it is challenging for other imputation approaches to distinguish the 4 cell types.

**FIGURE 3:**
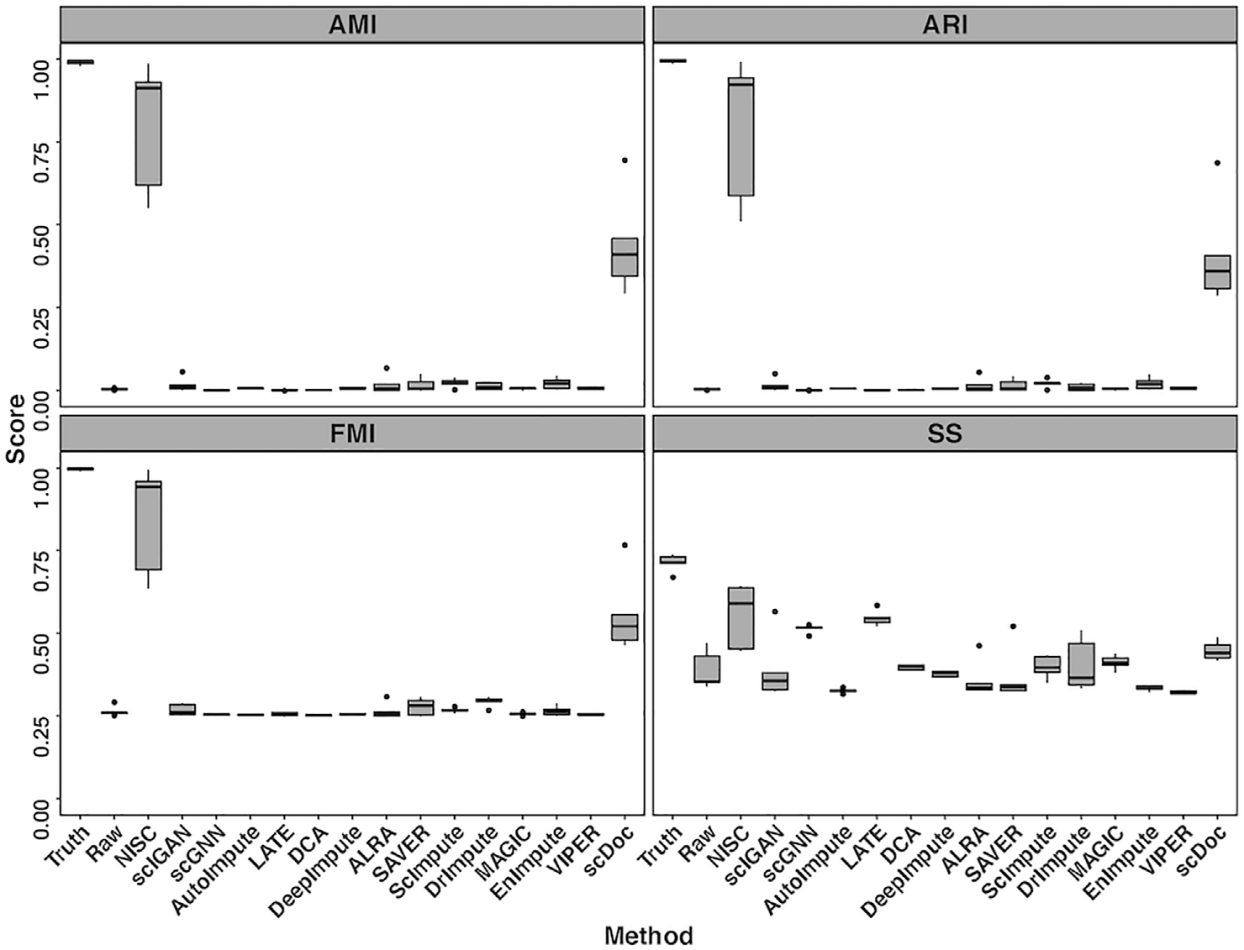
Boxplots of four evaluation measures, including Adjusted Mutual Information (AMI), Adjusted Rand Index (ARI), Fowlkes-Mallows Index (FMI), and Silhouette Score (SS), are calculated for comparing NISC and other imputation methods. Each dataset contains 800 genes and 1,000 cells in 4 cell types, with 90% sparsity, and is replicated 10 times. Detailed information about these measurements can be found in the supplementary materials.

Four evaluation metrics, including AMI, ARI, FMI, and SS are calculated on the visualization result for the simulated data in Figure 2. To consider the data uncertainty (even with the same parameter settings) in the simulation, we generated ten replicates of datasets under each setting. Figure 3 shows boxplots for four evaluation measures based on K-means clustering result of the t-SNE visualization. The boxplots of Leiden method are shown in Supplementary Figure S2. Higher values in measures indicate higher accuracy in cluster results. It is obvious that the performance of NISC surpasses all the existing imputation methods in clustering accuracy in this simulation study.

**FIGURE 4:**
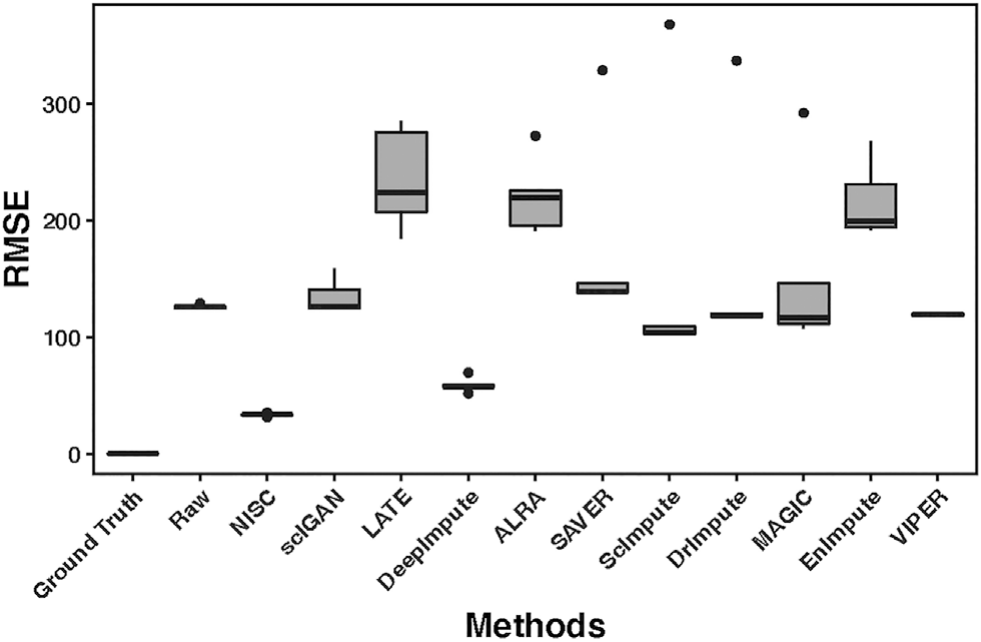
RMSE (root mean square error) boxplots for the raw input and imputed data by each method. The RMSE is calculated between the ground truth and either the raw or imputed values. The raw dataset contains 800 genes and 1,000 cells in 4 cell types, with 90% sparsity, and is replicated 10 times. Detailed information about the RMSE can be found in the supplementary materials.

High accuracy in cell type visualization does not necessarily mean the imputed values are close to the true values. We calculated RMSE (root mean square error, the detailed definition can be found in the supplementary materials) between the ground truth value and the corresponding imputed value by each method. Figure 4 shows boxplots of RMSE for 10 replicates of simulations. Compared with other imputation methods, the accuracy of NISC is highest, followed by DeepImpute, which is a neural network-based imputation method as well. Note: three imputation methods, DCA, AutoImpute and scGNN, are excluded from the RMSE plot as only highly variable genes are selected in these methods to perform imputation.

**FIGURE 5:**
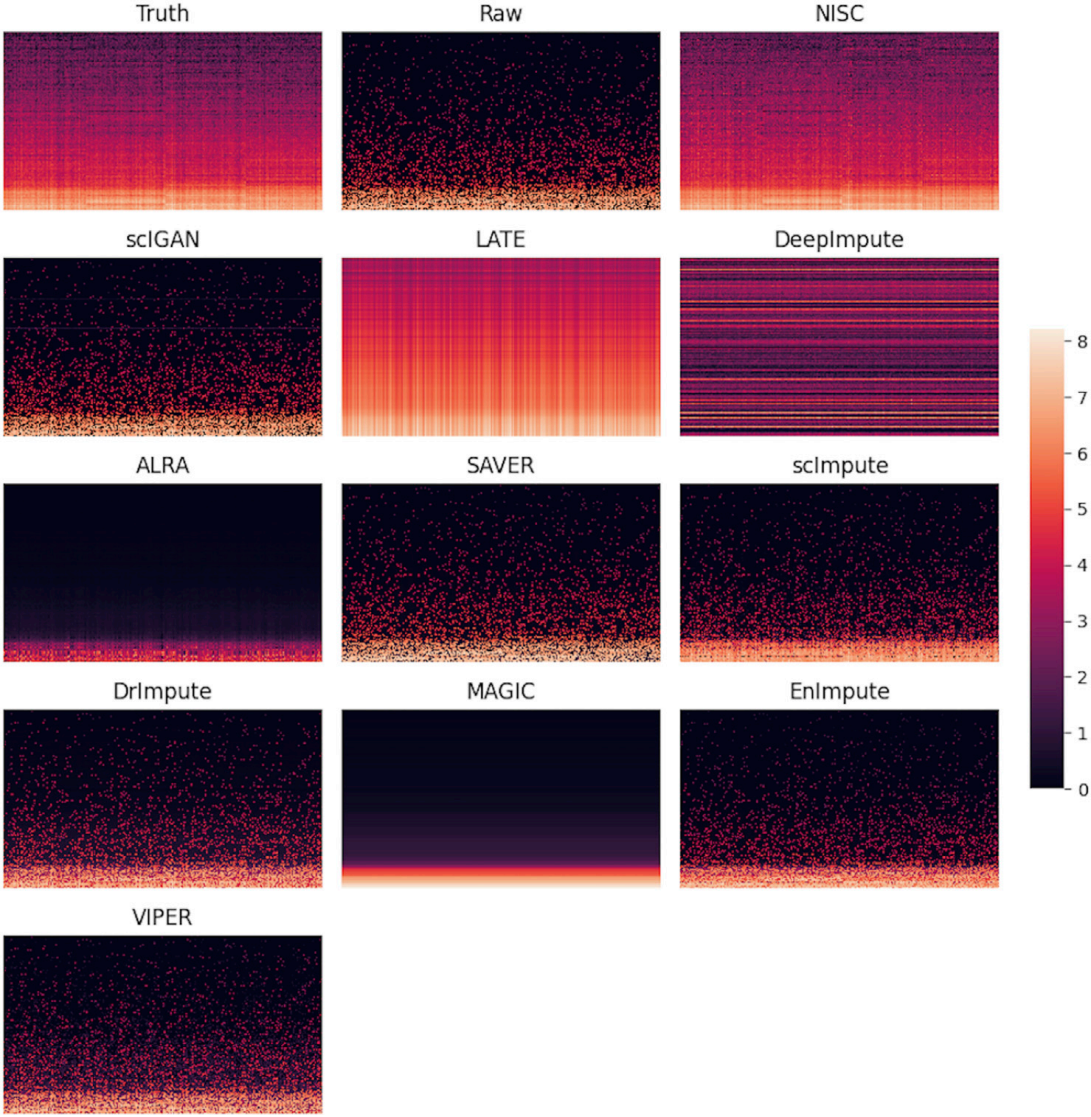
Heatmaps of ground truth, raw simulated data, and imputed data by various methods. The simulated raw dataset contains 800 genes and 1,000 cells in 4 cell types, with 90% sparsity. Each row in the heatmap represents a gene, while each column represents a cell. The color bar shows the magnitude of the logarithm of gene expression values.

A direct comparison in gene expression values among the ground truth, raw data, and imputed data can be found in the heatmap plot (Figure 5). It shows that NISC imputed values are closest to the ground truth and therefore this method shows great capability in correcting the dropout values, which confirms the promising result in data visualization in Figure 2. Again, three imputation methods DCA, AutoImpute, and scGNN, are excluded from the heatmap plot as only highly variable genes are selected in these methods to perform imputation.

A consistent conclusion can be obtained from UMAP plot (Supplementary Figure S3) for this dataset. We also examine the impact of a different sparsity level (80%) on the imputation for the simulated data with 4 cell types and 2 cell types, respectively. When the sparsity of the simulated data with 4 cell types is about 80%, the cell populations can be revealed clearly in several imputation methods (Supplementary Figure S4), and NISC is one of them. Then, we observe that the performance of all methods significantly decreases when dropout noise increases (Supplementary Figure S4 vs Figure 2). A consistent conclusion can be obtained for the 2 cell types. Supplementary Figure S5 shows an example of the t-SNE plot of 1,000 cells (in 2 cell types) and 800 genes with 80% sparsity. The cells are clearly separated into two groups/clusters by NISC, DeepImpute, DrImpute, EnImpute and scDoc, followed by scImpute, SAVER, and scIGAN.

For the case of 4 cell types with 10,000 cells, we only compared the deep-learning-based methods (Supplementary Figure S6). We noticed that the performances of three methods, NISC, DCA, and DeepImpute, are improved when the number of cells increases from 1,000 (Figure 2) to 10,000 (Supplementary Figure S6). The t-SNE plot in Supplementary Figure S6 still shows that NISC surpasses other deep-learning-based methods, followed by DCA and DeepImpute.

**Computational time**: Among the deep-learning-based methods, LATE is the fastest, and scIGAN is the slowest. Specifically, the order of the computational time for seven deep learning-based methods is: LATE < DeepImpute < DCA < NISC < AutoImpute < scGNN < scIGAN. We used High Performance Computer systems with 2894 MHz CPU, 5 cores, and 36 GB memory on each core. For a simulation dataset with 2,000 genes and 10,000 cells, it took about 10 min for LATE, 12 h scIGAN, and 50 min for NISC.

### 3.2 NISC Improves Visualization Clarity and Clustering Accuracy in Real scRNA-Seq Data

#### 3.2.1 Mouse Lung scRNA-Seq Data

**FIGURE 6:**
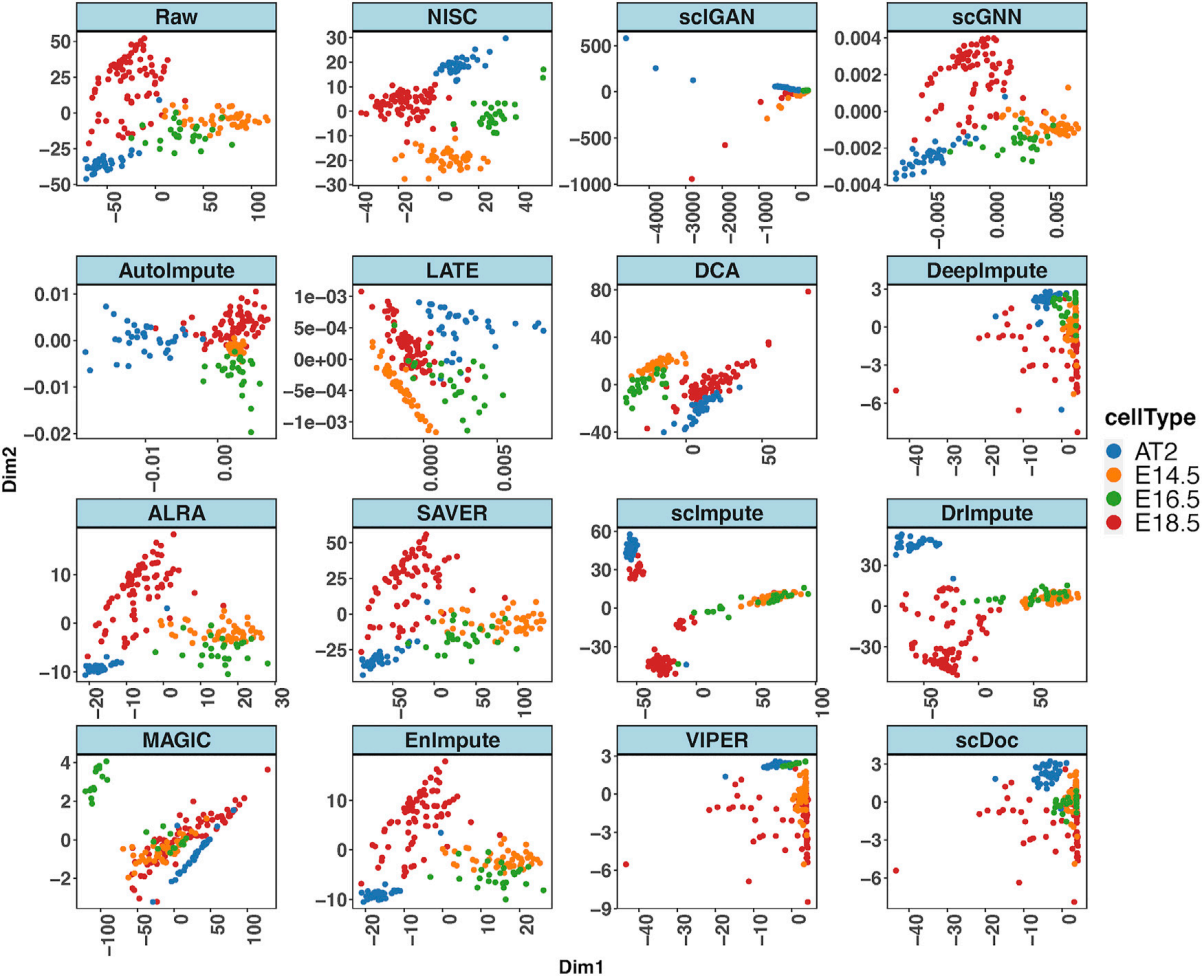
NISC recovers the cell types (E14.5, E16.5, E18.5, and AT2) in mouse lung data. PCA plots of the raw data and imputed data by various imputation methods. The sparsity of the data is 72.6%. Cells are colored by cell types, which are reported in the original publication.

We apply NISC and the compared methods on mouse lung scRNA-seq data (GSE52583) with 201 cells (Treutlein et al., 2014). Figure 6 shows PCA plots for NISC and other imputation methods. The denoised data by imputation of scGNN, AutoImpute, ALRA, SAVER, scImpute, DrImpute, scDoc and EnImpute show E14.5 and E16.5 are not separated well, although cell type AT2 and E18.5 can be identified. In addition, with imputation of MAGIC, E16.5 is successfully identified, but E18.5, E14.5, and AT2 are mixed. By DCA, the 4 cell types (E14.5, E16.5, E18.5, and AT2) are grouped into two clusters, with two types in each. For DeepImpute, scIGAN and VIPER, the 4 cell types are mixed together. It seems that NISC can assign the 4 cell types into four clusters more accurately.

**FIGURE 7:**
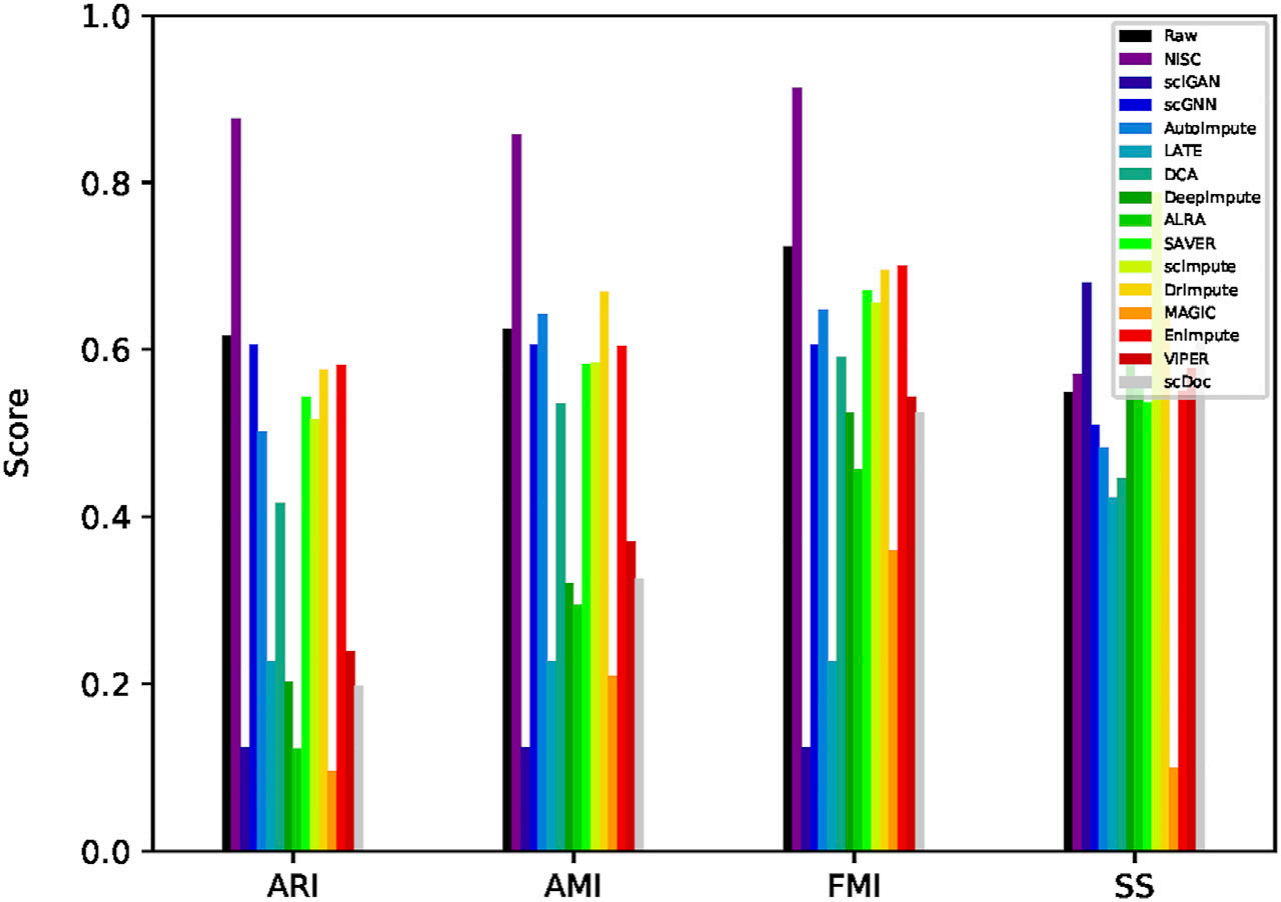
Evaluation of clustering accuracy on the mouse lung data. Four measurements, AMI, ARI, FMI, and SS, are calculated for the imputed and raw data. The definitions of the measurements can be found in the supplementary materials.

The evaluation matrices on the clustering for this dataset are also calculated (Figure 7). Though NISC result does not provide the tightest clusters (from Silhouette score), among all the imputation methods, it scores the highest consistently across three measures of clustering accuracy, which confirms the separation pattern in the visualization in Figure 6.

#### 3.2.2 Mouse Embryonic Data

We also apply NISC and the compared methods on scRNA-seq data of 92 mouse embryonic cells and 22,936 genes (GSE29087). The sparsity of the data is 83.04%. The cell types of this data set are reported in the original publication (Islam et al., 2011). We visualize the clustering result with t-SNE plots (Supplementary Figure S7), illustrating that, through NISC imputation, the 2 cell types, 48 mouse embryonic stem cells (ES) and 44 mouse embryonic fibroblasts (MEF), are separated, followed by DrImpute, DCA and scGNN. Through imputation of scIGAN, AutoImpute, LATE, ALRA, SAVER, MAGIC, EnImpute, SAVER, VIPER, and scDoc, the 2 cell types in this data are not separated well. With imputation of scGNN, DCA, and DrImpute, the 2 cell types are only somewhat separated. With scImpute, the cells are isolated into many tighter subclusters. In other words, some cells which should belong to the same cell type are scattered. The accuracy of clustering is assessed by four evaluation measures. Though NISC result does not provide the tightest clusters (from Silhouette score), among all the methods compared here, NISC is superior to others in terms of cluster accuracy ARI, AMI, and FMI. It improves the cluster results on original raw data.

#### 3.2.3 Human Lung Adenocarcinoma Data

**FIGURE 8:**
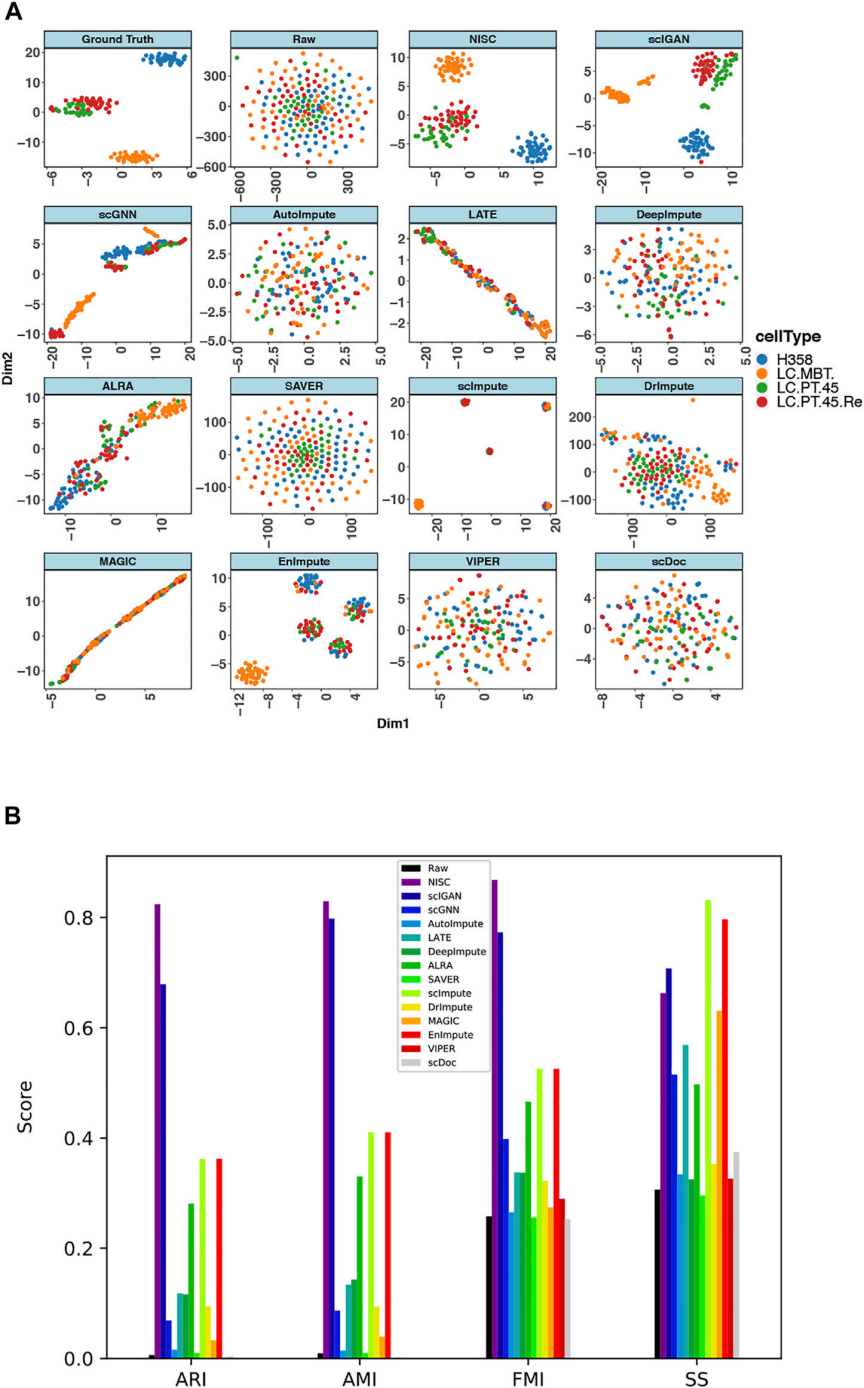
NISC recovers the cell types in lung adenocarcinoma data (GSE69405) **(A)** plots of t-SNE components 1 and 2 derived from raw data, imputed data using NISC and other imputation methods. With additional zeros the sparsity of the data is 80%. Cells are colored by cell types, which are reported in the original publication **(B)** Bar plots of evaluation of cluster accuracy on the raw and imputed data. Four measurements, AMI, ARI, FMI, and SS, are calculated for the imputed and raw data. The definitions of the measurements can be found in the supplementary.

The above real scRNA-seq datasets do not have ground truth, since usually it is challenging to obtain the ground truth for real scRNA-seq data. Alternatively, it will be convincing to evaluate the performance of the imputation approaches if we use a real scRNA-seq dataset with low sparsity and distinct cell types and set it to be the ground truth data for evaluations. For this purpose, we apply the imputation methods on lung adenocarcinoma data (GSE69405) that profiles the gene expression of single cancer cells with TPM (normalization by transcripts per million) measurements (Soneson and Robinson, 2018). These cancer cells are originally from lung adenocarcinoma patient-derived xenograft (PDX) tumors, including four types, H358 human lung cancer cells (H358), cancer cells in PDX from primary tumors (LC-PT-45), an additional batch of PDX cells (LC-Pt-45-Re), and PDX cells for another lung cancer case (LC-MBT-15). This data set contains 176 cells, and the sparsity of the data is relatively low (46%). The cell types in this data can be clearly identified in the original data without imputation (Figure 8A). Therefore, we set the original data to be the ground truth. Following the method in (Arisdakessian et al., 2019) to generate noisy data, similarly, we mask the low-noise data by randomly changing some non-zeros to zeros so that the sparsity of the data is increased to 80% and the synthesized dataset here is termed as raw data.

T-SNE plots (Figure 8A) of the synthesized data show that NISC successfully recovers the cell types of the original data through imputing the sparse raw data. However, other imputation methods result in either one big cluster (i.e., all cells are mixed together) or several tight clusters, but each with two or more different cell types. A consistent conclusion can be obtained in evaluation plots (Figure 8B). Though the cells are not separately into tight clusters in NISC data, this method results in the highest cluster accuracy, considering the actual cell type status.

## 4 Discussion

NISC is a data-driven method and does not require any prior knowledge. Real data and simulated data show that NISC can impute the dropouts in the scRNA-seq data, improving the accuracy of cell type clustering. Four performance measures were calculated to evaluate the clustering accuracy for the imputed data by various imputation methods. RMSE, which measures the distance between true (if available) and imputed values, was also calculated. Generally, compared with other existing estimation methods, NISC has a lower RMSE and a higher score in the evaluation measures of clustering accuracy.

NISC is an unsupervised neural network-based imputation method with autoencoder techniques implemented. Compared with other neural network-based methods, we investigated how different loss functions affect the imputation results. We developed a novel loss function weighted by Michaelis-Menten kinetics (MMK) and investigated its difference and standard mean square error (MSE) loss. Fig. S1 shows that the MMK loss can achieve more effective imputation under the sparse simulation setting, while by regular MSE the loss function is less effective. In addition, we add L2 regularization and dropout regularization to the model (Cortes et al., 2012) to avoid overfitting when denoising the input data. This is the first time the two regularizations are implemented simultaneously in the autoencoder model to impute scRNA-seq data.

An effective neural network for imputation requires sufficient neurons in the network. Due to many genes in scRNA-seq studies, GPUs are recommended for NISC to speed up the training process of the autoencoder network. NISC imputation is not suitable for some types of data which lose Michaelis-Menten kinetics, such as 10x Genomics data (Andrews and Hemberg, 2019), and some normalized data, for example, RPKM (Reads per kilo base per million mapped reads) or FPKM (Fragments Per Kilobase Million) (Lytal et al., 2020). However, TPM normalization is applicable as it maintains the data structure of the original gene expressions (Li and Li, 2018).

## Data Availability

The original contributions presented in the study are publicly available. This data can be found here: github.com/anlingUA/NISC.

## References

[B1] Andrews T. S. Hemberg M. (2019). M3Drop: Dropout-Based Feature Selection for scRNASeq. Bioinformatics 35 (16), 2865–2867. 10.1093/bioinformatics/bty1044 30590489 PMC6691329

[B2] Angerer P. Simon L. Tritschler S. Wolf F. A. Fischer D. Theis F. J. (2017). Single Cells Make Big Data: New Challenges and Opportunities in Transcriptomics. Curr. Opin. Syst. Biol. 4, 85–91. 10.1016/j.coisb.2017.07.004

[B3] Anowar F. Sadaoui S. Selim B. (2021). Conceptual and empirical comparison of dimensionality reduction algorithms (pca, kpca, lda, mds, svd, lle, isomap, le, ica, t-sne). Comp. Sci. Rev. 40, 100378. 10.1016/j.cosrev.2021.100378

[B4] Arisdakessian C. Poirion O. Yunits B. Zhu X. Garmire L. X. (2019). DeepImpute: an Accurate, Fast, and Scalable Deep Neural Network Method to Impute Single-Cell RNA-Seq Data. Genome Biol. 20 (1), 211–214. 10.1186/s13059-019-1837-6 31627739 PMC6798445

[B5] Badsha M. B. Li R. Liu B. Li Y. I. Xian M. Banovich N. E. (2020). Imputation of Single-Cell Gene Expression with an Autoencoder Neural Network. Quant Biol. 8 (1), 78–94. 10.1007/s40484-019-0192-7 32274259 PMC7144625

[B6] Becht E. McInnes L. Healy J. Dutertre C.-A. Kwok I. W. H. Ng L. G. (2019). Dimensionality Reduction for Visualizing Single-Cell Data Using UMAP. Nat. Biotechnol. 37 (1), 38–44. 10.1038/nbt.4314 30531897

[B7] Bengtsson M. Ståhlberg A. Rorsman P. Kubista M. (2005). Gene Expression Profiling in Single Cells from the Pancreatic Islets of Langerhans Reveals Lognormal Distribution of mRNA Levels. Genome Res. 15 (10), 1388–1392. 10.1101/gr.3820805 16204192 PMC1240081

[B8] Blondel V. D. Guillaume J.-L. Lambiotte R. Lefebvre E. (2008). Fast Unfolding of Communities in Large Networks. J. Stat. Mech. 2008 (10), P10008. 10.1088/1742-5468/2008/10/p10008

[B9] Brennecke P. Anders S. Kim J. K. Kołodziejczyk A. A. Zhang X. Proserpio V. (2013). Accounting for Technical Noise in Single-Cell RNA-Seq Experiments. Nat. Methods 10 (11), 1093–1095. 10.1038/nmeth.2645 24056876

[B10] Chen M. Zhou X. (2018). VIPER: Variability-Preserving Imputation for Accurate Gene Expression Recovery in Single-Cell RNA Sequencing Studies. Genome Biol. 19 (1), 1–15. 10.1186/s13059-018-1575-1 30419955 PMC6233584

[B11] Cortes C. Mohri M. Rostamizadeh A. (2012). L2 Regularization for Learning Kernels. arXiv preprint arXiv:1205.2653.

[B12] Eraslan G. Simon L. M. Mircea M. Mueller N. S. Theis F. J. (2019). Single-cell RNA-Seq Denoising Using a Deep Count Autoencoder. Nat. Commun. 10 (1), 1–14. 10.1038/s41467-018-07931-2 30674886 PMC6344535

[B13] Eweda E. Macchi O. (1984). Convergence of an Adaptive Linear Estimation Algorithm. IEEE Trans. Automat. Contr. 29 (2), 119–127. 10.1109/tac.1984.1103463

[B14] Gong W. Kwak I. Y. Pota P. Koyano-Nakagawa N. Garry D. J. (2018). DrImpute: Imputing Dropout Events in Single Cell RNA Sequencing Data. BMC bioinformatics 19 (1), 1–10. 10.1186/s12859-018-2226-y 29884114 PMC5994079

[B15] Gordon A. J. Satory D. Halliday J. A. Herman C. (2015). Lost in Transcription: Transient Errors in Information Transfer. Curr. Opin. Microbiol. 24, 80–87. 10.1016/j.mib.2015.01.010 25637723 PMC4380820

[B16] Grønbech C. H. Vording M. F. Timshel P. N. Sønderby C. K. Pers T. H. Winther O. (2020). scVAE: Variational Auto-Encoders for Single-Cell Gene Expression Data. Bioinformatics 36 (16), 4415–4422. 10.1093/bioinformatics/btaa293 32415966

[B17] Hinton G. E. Salakhutdinov R. R. (2006). Reducing the Dimensionality of Data with Neural Networks. Science 313 (5786), 504–507. 10.1126/science.1127647 16873662

[B18] Huang M. Wang J. Torre E. Dueck H. Shaffer S. Bonasio R. (2018). SAVER: Gene Expression Recovery for Single-Cell RNA Sequencing. Nat. Methods 15 (7), 539–542. 10.1038/s41592-018-0033-z 29941873 PMC6030502

[B19] Islam S. Kjällquist U. Moliner A. Zajac P. Fan J.-B. Lönnerberg P. (2011). Characterization of the Single-Cell Transcriptional Landscape by Highly Multiplex RNA-Seq. Genome Res. 21 (7), 1160–1167. 10.1101/gr.110882.110 21543516 PMC3129258

[B20] Johnson K. A. Goody R. S. (2011). The Original Michaelis Constant: Translation of the 1913 Michaelis-Menten Paper. Biochemistry 50 (39), 8264–8269. 10.1021/bi201284u 21888353 PMC3381512

[B21] Kin T. Tsuda K. Asai K. (2002). Marginalized Kernels for RNA Sequence Data Analysis. Genome Inform. 13, 112–122. 14571380

[B22] Kobak D. Berens P. (2019). The Art of Using T-SNE for Single-Cell Transcriptomics. Nat. Commun. 10 (1), 1–14. 10.1038/s41467-019-13056-x 31780648 PMC6882829

[B23] Li W. V. Li J. J. (2018). An Accurate and Robust Imputation Method scImpute for Single-Cell RNA-Seq Data. Nat. Commun. 9 (1), 997–999. 10.1038/s41467-018-03405-7 29520097 PMC5843666

[B24] Lin P. Troup M. Ho J. W. (2017). CIDR: Ultrafast and Accurate Clustering through Imputation for Single-Cell RNA-Seq Data. Genome Biol. 18 (1), 59–11. 10.1186/s13059-017-1188-0 28351406 PMC5371246

[B25] Linderman G. C. Zhao J. Kluger Y. (2018). Zero-preserving Imputation of scRNA-Seq Data Using Low-Rank Approximation. BioRxiv, 397588.

[B26] Lopez R. Regier J. Cole M. B. Jordan M. I. Yosef N. (2018). Deep Generative Modeling for Single-Cell Transcriptomics. Nat. Methods 15 (12), 1053–1058. 10.1038/s41592-018-0229-2 30504886 PMC6289068

[B27] Lytal N. Ran D. An L. (2020). Normalization Methods on Single-Cell RNA-Seq Data: an Empirical Survey. Front. Genet. 11, 41. 10.3389/fgene.2020.00041 32117453 PMC7019105

[B28] Mao X. J. Shen C. Yang Y. B. (2016). Image Restoration Using Convolutional Auto-Encoders with Symmetric Skip Connections. arXiv preprint arXiv:1606.08921.

[B29] Murtagh F. Contreras P. (2017). Algorithms for Hierarchical Clustering: an Overview, II. Wiley Interdiscip. Rev. Data Mining Knowledge Discov. 7 (6), e1219. 10.1002/widm.1219

[B30] Na S. Xumin L. Yong G. (2010). “Research on K-Means Clustering Algorithm: An Improved K-Means Clustering Algorithm,” in Proceeding of the 2010 Third International Symposium on intelligent information technology and security informatics, Jian, China, April 2010 (IEEE), 63–67. 10.1109/iitsi.2010.74

[B31] Nemec A. F. L. Brinkhurst R. O. (1988). The Fowlkes-Mallows Statistic and the Comparison of Two Independently Determined Dendrograms. Can. J. Fish. Aquat. Sci. 45 (6), 971–975. 10.1139/f88-119

[B32] Ng A. Y. (2004). “Feature Selection, L 1 vs. L 2 Regularization, and Rotational Invariance,” in Proceedings of the twenty-first international conference on Machine learning, July 2004, 78.

[B33] Pierson E. Yau C. (2015). ZIFA: Dimensionality Reduction for Zero-Inflated Single-Cell Gene Expression Analysis. Genome Biol. 16 (1), 1–10. 10.1186/s13059-015-0805-z 26527291 PMC4630968

[B34] Ran D. Zhang S. Lytal N. An L. (2020). scDoc: Correcting Drop-Out Events in Single-Cell RNA-Seq Data. Bioinformatics 36 (15), 4233–4239. 10.1093/bioinformatics/btaa283 32365169

[B35] Reiter M. Kirchner B. Muller H. Holzhauer C. Mann W. Pfaffl M. W. (2011). Quantification Noise in Single Cell Experiments. Nucleic Acids Res. 39 (18), e124. 10.1093/nar/gkr505 21745823 PMC3185419

[B36] Romano S. Bailey J. Nguyen V. Verspoor K. (2014). “Standardized Mutual Information for Clustering Comparisons: One Step Further in Adjustment for Chance,” in Proceedings of the International Conference on Machine Learning, Aug 2021, 1143–1151.

[B37] Rousseeuw P. J. (1987). Silhouettes: a Graphical Aid to the Interpretation and Validation of Cluster Analysis. J. Comput. Appl. Math. 20, 53–65. 10.1016/0377-0427(87)90125-7

[B38] Shao L. Yan R. Li X. Liu Y. (2013). From Heuristic Optimization to Dictionary Learning: A Review and Comprehensive Comparison of Image Denoising Algorithms. IEEE Trans. Cybern 44 (7), 1001–1013. 10.1109/TCYB.2013.2278548 24002014

[B39] Skinnider M. A. Squair J. W. Foster L. J. (2019). Evaluating Measures of Association for Single-Cell Transcriptomics. Nat. Methods 16 (5), 381–386. 10.1038/s41592-019-0372-4 30962620

[B40] Soneson C. Robinson M. D. (2018). Bias, Robustness and Scalability in Single-Cell Differential Expression Analysis. Nat. Methods 15 (4), 255–261. 10.1038/nmeth.4612 29481549

[B41] Srivastava N. Hinton G. Krizhevsky A. Sutskever I. Salakhutdinov R. (2014). Dropout: a Simple Way to Prevent Neural Networks from Overfitting. J. machine Learn. Res. 15 (1), 1929–1958. 10.5555/2627435.2670313

[B42] Steinley D. (2004). Properties of the Hubert-Arable Adjusted Rand Index. Psychol. Methods 9 (3), 386–396. 10.1037/1082-989x.9.3.386 15355155

[B43] Sun S. Liu Y. Shang X. (2019). “Deep Generative Autoencoder for Low-Dimensional Embeding Extraction from Single-Cell RNAseq Data,” in Proceedings of the 2019 IEEE International Conference on Bioinformatics and Biomedicine (BIBM), San Diego, CA, USA, Nov. 2019 (IEEE), 1365–1372. 10.1109/bibm47256.2019.8983289

[B44] Talwar D. Mongia A. Sengupta D. Majumdar A. (2018). AutoImpute: Autoencoder Based Imputation of Single-Cell RNA-Seq Data. Sci. Rep. 8 (1), 1–11. 10.1038/s41598-018-34688-x 30397240 PMC6218547

[B45] Tangherloni A. Ricciuti F. Besozzi D. Liò P. Cvejic A. (2021). Analysis of Single-Cell RNA Sequencing Data Based on Autoencoders. BMC bioinformatics 22 (1), 309–327. 10.1186/s12859-021-04150-3 34103004 PMC8186186

[B46] Traag V. A. Waltman L. Van Eck N. J. (2019). From Louvain to Leiden: Guaranteeing Well-Connected Communities. Sci. Rep. 9 (1), 1–12. 10.1038/s41598-019-41695-z 30914743 PMC6435756

[B47] Tracy S. Yuan G. C. Dries R. (2019). RESCUE: Imputing Dropout Events in Single-Cell RNA-Sequencing Data. BMC bioinformatics 20 (1), 1–11. 10.1186/s12859-019-2977-0 31299886 PMC6624880

[B48] Treutlein B. Brownfield D. G. Wu A. R. Neff N. F. Mantalas G. L. Espinoza F. H. (2014). Reconstructing Lineage Hierarchies of the Distal Lung Epithelium Using Single-Cell RNA-Seq. Nature 509 (7500), 371–375. 10.1038/nature13173 24739965 PMC4145853

[B49] Van Dijk D. Sharma R. Nainys J. Yim K. Kathail P. Carr A. J. (2018). Recovering Gene Interactions from Single-Cell Data Using Data Diffusion. Cell 174 (3), 716–729. 10.1016/j.cell.2018.05.061 29961576 PMC6771278

[B50] Vincent P. Larochelle H. Lajoie I. Bengio Y. Manzagol P. A. Bottou L. (2010). Stacked Denoising Autoencoders: Learning Useful Representations in a Deep Network with a Local Denoising Criterion. J. machine Learn. Res. 11 (12), 3371–3408.

[B51] Wang D. Gu J. (2018). VASC: Dimension Reduction and Visualization of Single-Cell RNA-Seq Data by Deep Variational Autoencoder. Genomics, proteomics & bioinformatics 16 (5), 320–331. 10.1016/j.gpb.2018.08.003 PMC6364131 30576740

[B52] Wang J. Ma A. Chang Y. Gong J. Jiang Y. Qi R. (2021). scGNN Is a Novel Graph Neural Network Framework for Single-Cell RNA-Seq Analyses. Nat. Commun. 12 (1), 1–11. 10.1038/s41467-021-22197-x 33767197 PMC7994447

[B53] Xing C. Ma L. Yang X. (2016). Stacked Denoise Autoencoder Based Feature Extraction and Classification for Hyperspectral Images. J. Sensors 2016, 1–10. 10.1155/2016/3632943

[B54] Xu Y. Zhang Z. You L. Liu J. Fan Z. Zhou X. (2020). scIGANs: Single-Cell RNA-Seq Imputation Using Generative Adversarial Networks. Nucleic Acids Res. 48 (15), e85. 10.1093/nar/gkaa506 32588900 PMC7470961

[B55] Zappia L. Phipson B. Oshlack A. (2017). Splatter: Simulation of Single-Cell RNA Sequencing Data. Genome Biol. 18 (1), 1–15. 10.1186/s13059-017-1305-0 28899397 PMC5596896

[B56] Zhang L. Zhang S. (2018). Comparison of Computational Methods for Imputing Single-Cell RNA-Sequencing Data. IEEE/ACM Trans. Comput. Biol. Bioinform. 17 (2), 174–389. 10.1109/tcbb.2018.2848633 29994128

[B57] Zhang X.-F. Ou-Yang L. Yang S. Zhao X.-M. Hu X. Yan H. (2019). EnImpute: Imputing Dropout Events in Single-Cell RNA-Sequencing Data via Ensemble Learning. Bioinformatics 35 (22), 4827–4829. 10.1093/bioinformatics/btz435 31125056

