# Supplementary material for: NISC: Neural Network-Imputation for Single-Cell RNA Sequencing and Cell Type Clustering
Source: Front Genet. 2022 May 3;13:847112. doi: 10.3389/fgene.2022.847112 (PMC9110639; doi:10.3389/fgene.2022.847112)
Supplement: Supplementary file 1 [file DataSheet1.PDF]

Supplementary Material

1 Loss Functions

We compared Michaelis-Menten kinetics (MMK) weighted loss function with a typical loss function, mean squared error (MSE) loss, using simulation studies. MSE loss is the loss function of the neural network by minimizing the mean distance between the predicted imputation and the observed gene expression values:

$$MSE\ Loss = \frac{\sum_{i=1}^{m}\sum_{j=1}^{n}\left(log(\hat{y}_{ij}) - \log\,(y_{ij})\right)^2}{m \times n}$$

where y_{ij} is the observed gene expression for gene i in cell j. $\hat{y}_{ij}$is the predicted value of imputation. Throughout the text, the function "*log*" is the natural logarithm.

MMK loss is the loss function that we proposed:

$$MMK\ Loss = \sum_{i=1}^{m}\sum_{j=1}^{n}(1 - P_{ij}) \cdot \left(log(\hat{y}_{ij}) - log(y_{ij})\right)^2 + \alpha \cdot \parallel \beta \parallel_2$$

where $P_{ij} = 1 - \frac{S_{ij}}{K_M + S_{ij}}$, and S_{ij} is the observed gene expression level of gene *i* in cell *j*, and K_M is the Michaelis constant (Johnson and Goody, 2011).

According to the visualization result from a simulation study (Fig.S1), MSE loss shows lack of accuracy in distinguishing the cell types as compared to MMK loss, which implies that MMK loss increases the power in cell types identification.

2 Evaluation Metrics

Mean Square Error (MSE), Root Mean Square Error ($RMSE$) and Mean Absolute Deviation (MAD) on log transformed counts were calculated to evaluate the performance of imputation methods (Badsha et al., 2020). MSE and $RMSE$ are metrics to measure the mean differences between the imputed/raw values and the ground truth values (Skinnider et al., 2019). X_{ij} denotes the gene expresson level of ground truth for gene i in cell j. $\hat{X}_{ij}$ referrers to the imputed/raw values for the same gene. There are m genes in each cell and n cells in total. Similarly, MAD measures the mean distance between the imputed/raw gene expression and the ground truth across all genes and cells.

$$MSE = \frac{\sum_{i=1}^{m}\sum_{j=1}^{n}(\hat{X}_{ij} - X_{ij})^2}{m \times n}$$

$$RMSE = \sqrt{\frac{\sum_{i=1}^{m}\sum_{j=1}^{n}(\hat{X}_{ij} - X_{ij})^2}{m \times n}}$$

$$MAD = \frac{\sum_{i=1}^{m}\sum_{j=1}^{n} | \hat{X}_{ij} - X_{ij}|}{m \times n}$$

Additionally, we use four other evaluation metrics to evaluate the performance of the cell type clusters, including Adjusted mutual information (AMI) (Romano et al., 2014), Adjusted Rand index (ARI) (Steinley, 2004), Fowlkes-Mallows index (FMI) (Nemec & Brinkhurst, 1988), and Silhouette Coefficient (SC) (Rousseeuw, 1987). Adjusted Rand index (ARI) is a function that measures the similarity between the ground truth assignments and the clustering algorithm assignments of the same data with chance normalization:

$$ARI = \frac{RI - E[RI]}{\max(RI) - E[RI]}$$

where $RI = \frac{a+b}{c}$. In this formula, a is the number of pairs of elements that are in the same set in ground truth and in the same set in clustering result; b is the number of pairs of elements that are in different sets in ground truth and in different sets in clustering; c is the total number of possible pairs in the data set.

Adjusted mutual information (AMI) is a function that measures the normalized mutual information between ground truth and clustering assignments. The following formula shows how to calculate the mutual information (MI) between two classes U and V:

$$MI(U,V) = \sum_{i=1}^{u}\sum_{j=1}^{v} P(i,j) log \frac{P(i,j)}{P(i)P(j)}$$

where $P(i,j)$ is the probability that an object picked at random falls into both classes U and V. $P(i)$ is the probability that an object belongs to class U. $P(j)$ is the probability that an object picked belongs to class V. AMI is defined as:

$$AMI(U,V) = \frac{MI(U,V) - E(MI(U,V))}{\max(\{H(U),H(V)\}) - E(MI(U,V))}$$

where $H(U)$ and $H(V)$ are entropy of U and V, respectively.

The Fowlkes-Mallows score (FMI) is a metric defined as the geometric mean of the pairwise precision:

$$FMI = \sqrt{\frac{TP}{TP + FP} \cdot \frac{TP}{TP + FN}}$$

where TP is number of the true positives, FP is false positives, and FN is the false negatives.

The Silhouette Coefficient SC is a metric used to measure the goodness of a clustering technique. It is calculated by the mean intra-cluster distance and the mean inter-cluster distance. SC is composed of two scores d_{intra} and d_{inter}.

$$SC = \frac{d_{inter} - d_{intra}}{\max\,(d_{intra}\,,\ d_{inter})}$$

where d_{intra} is the mean intra-cluster distance (i.e., the average distance between each point within a cluster) and d_{inter} is the mean inter-cluster distance (i.e., the average distance between all clusters).

3 Supplementary Figures

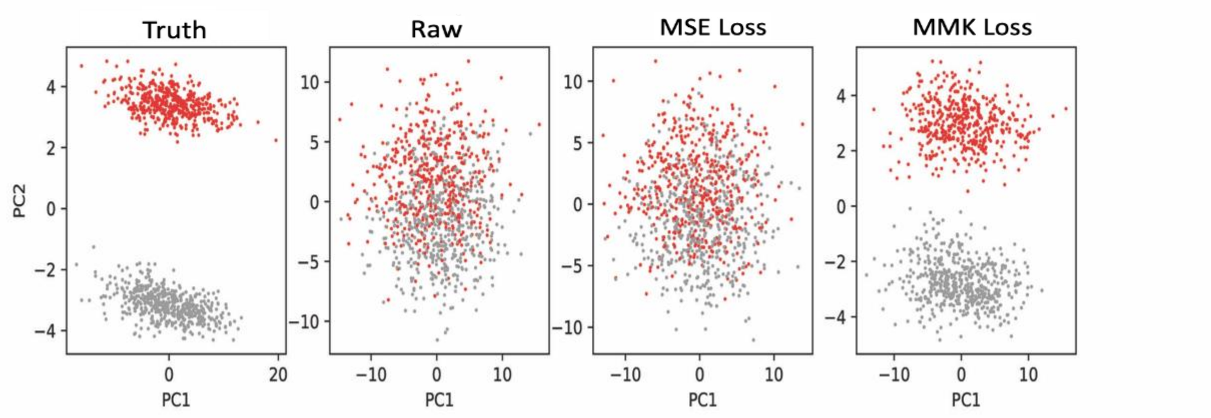

Supplementary Figure 1. Weighted Loss function is necessary for identifying cell types. Plots shows principal components 1 and 2 derived from simulated data without dropout (i.e., truth), with dropout (i.e., raw), with dropout denoised using autoencoder network with MSE loss and MMK loss, from left to right. The simulated data contains 800 genes and 1,000 cells, and the sparsity of the data is 70%.

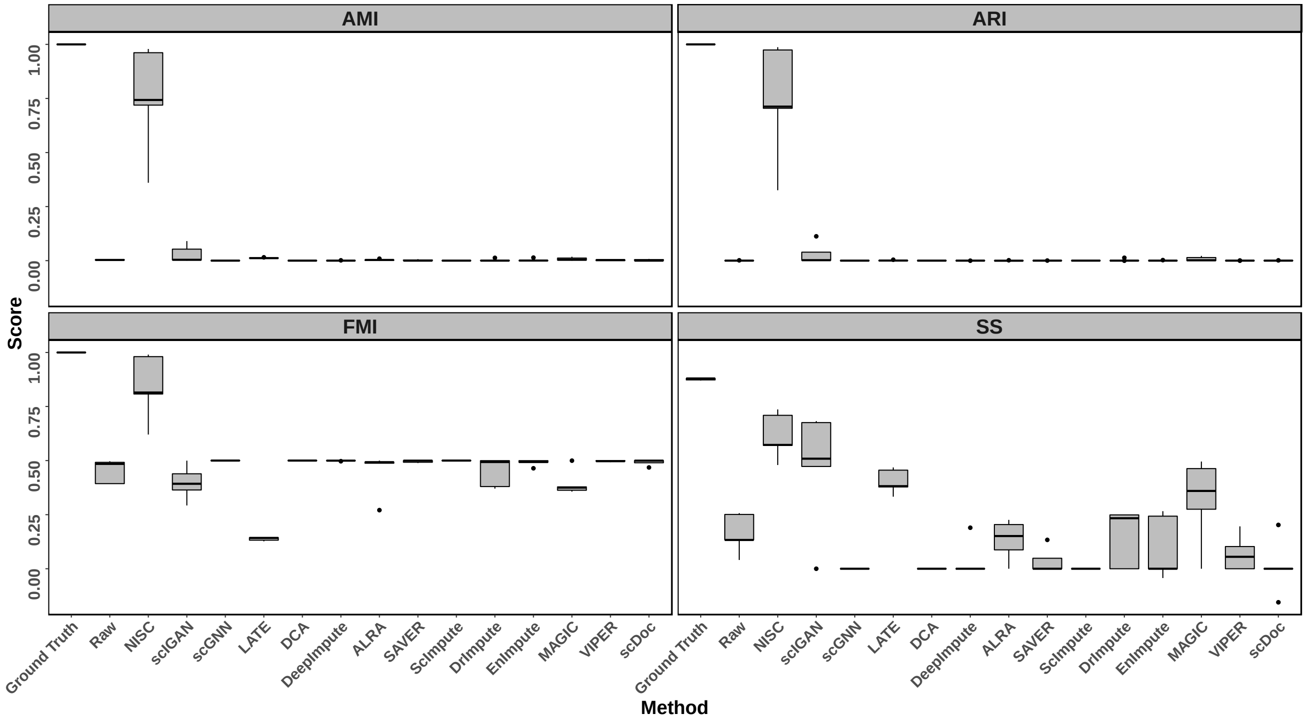

Supplementary Figure 2. Boxplots of four evaluation measures, including Adjusted Mutual Information (AMI), Adjusted Rand Index (ARI), Fowlkes-Mallows Index (FMI), and Silhouette Score (SS), are calculated for comparing NISC and other existing imputation methods. The details of the imputation methods can be found in the Introduction section in the main text. Each dataset contains 800 genes and 1,000 cells in four cell types, with 90% sparsity, and is replicated 10 times. The Leiden clustering algorithm is applied to the two-dimensional t-SNE (t-distributed stochastic neighbor embedding) plot.

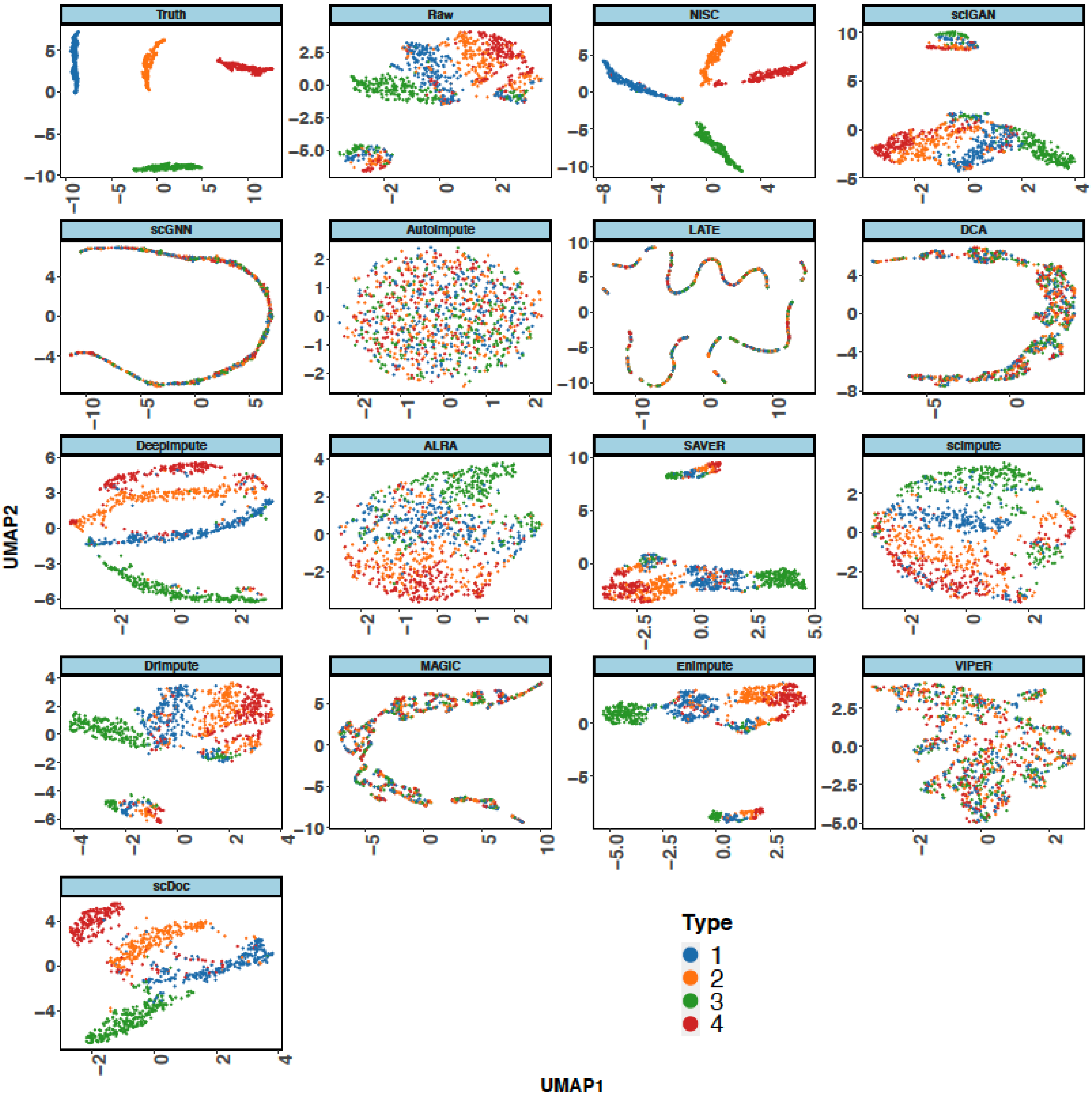

Supplementary Figure 3. UMAP (Uniform Manifold Approximation) plots of simulated data with 800 genes and 1,000 cells are drawn for ground truth, with dropout (i.e., raw), and imputed data using NISC and compared imputation methods. The details of the imputation methods can be found in the Introduction section in the main text. The simulated data contain 90% sparsity. Cells are colored by cell types.

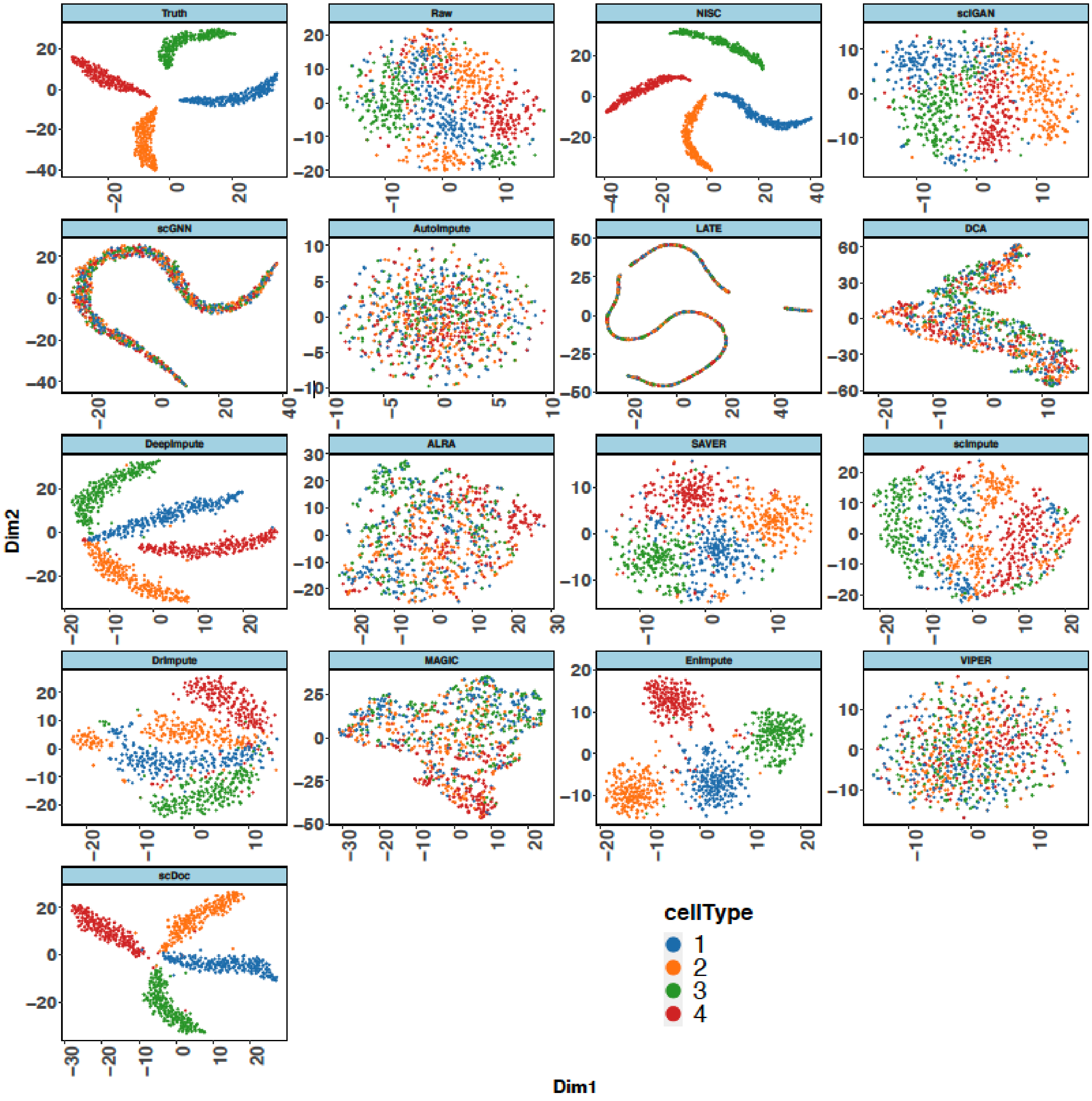

Supplementary Figure 4. t-SNE plot of simulated data with 800 genes and 1,000 cells are drawn for ground truth, with dropout (i.e., raw), and imputed data using NISC and compared imputation methods. The details of the imputation methods can be found in the Introduction section in the main text. The simulated data contain 80% sparsity. Cells are colored by cell types.

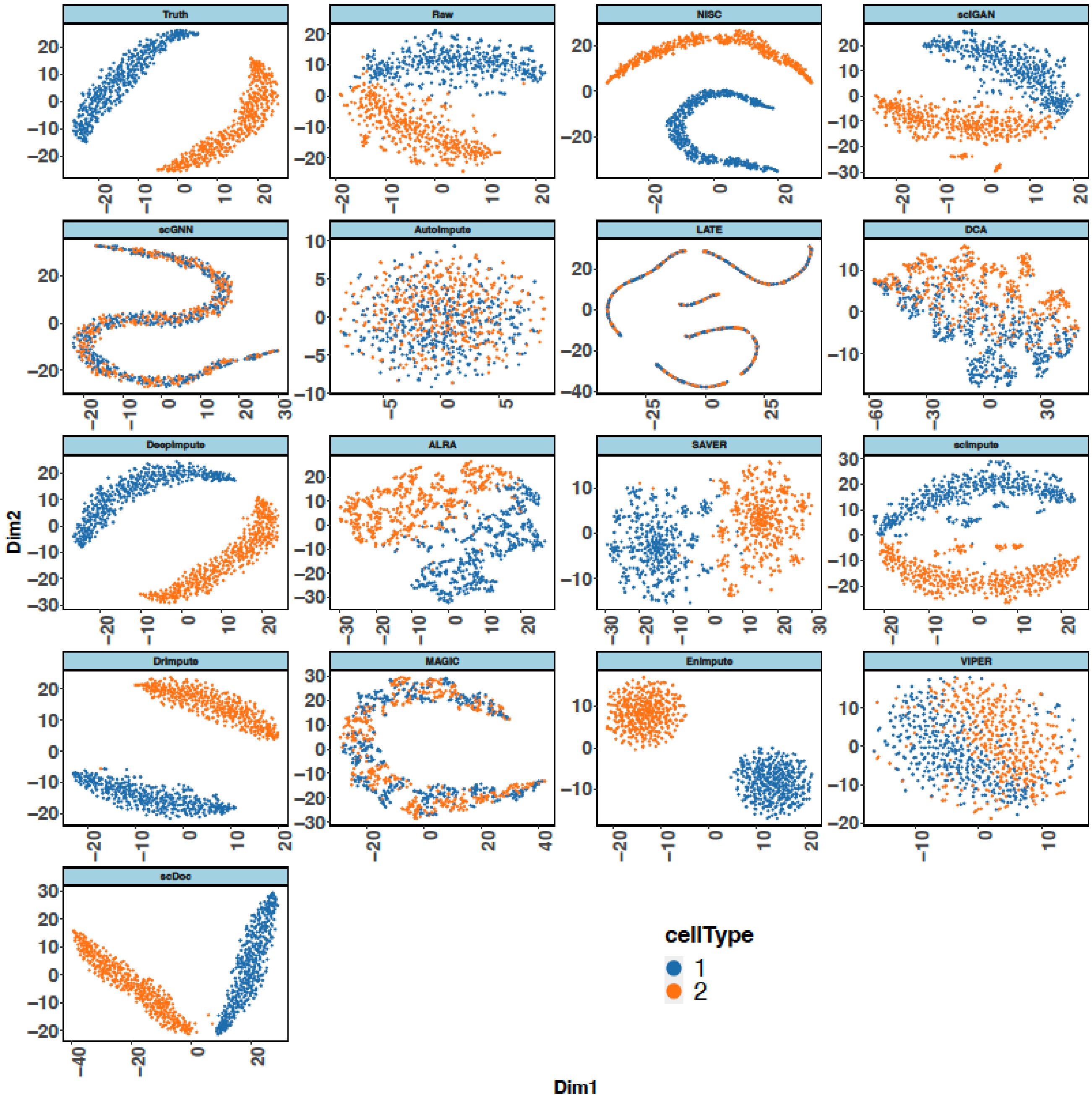

Supplementary Figure 5. t-SNE plots of the first two components are calculated for the simulated ground true data, raw data and imputed data using NISC and compared imputation methods. The details of the imputation methods can be found in the Introduction section in the main text. The dataset contains 800 genes and 1,000 cells in four cell types, with 80% sparsity. Cells are colored by cell types as indicated.

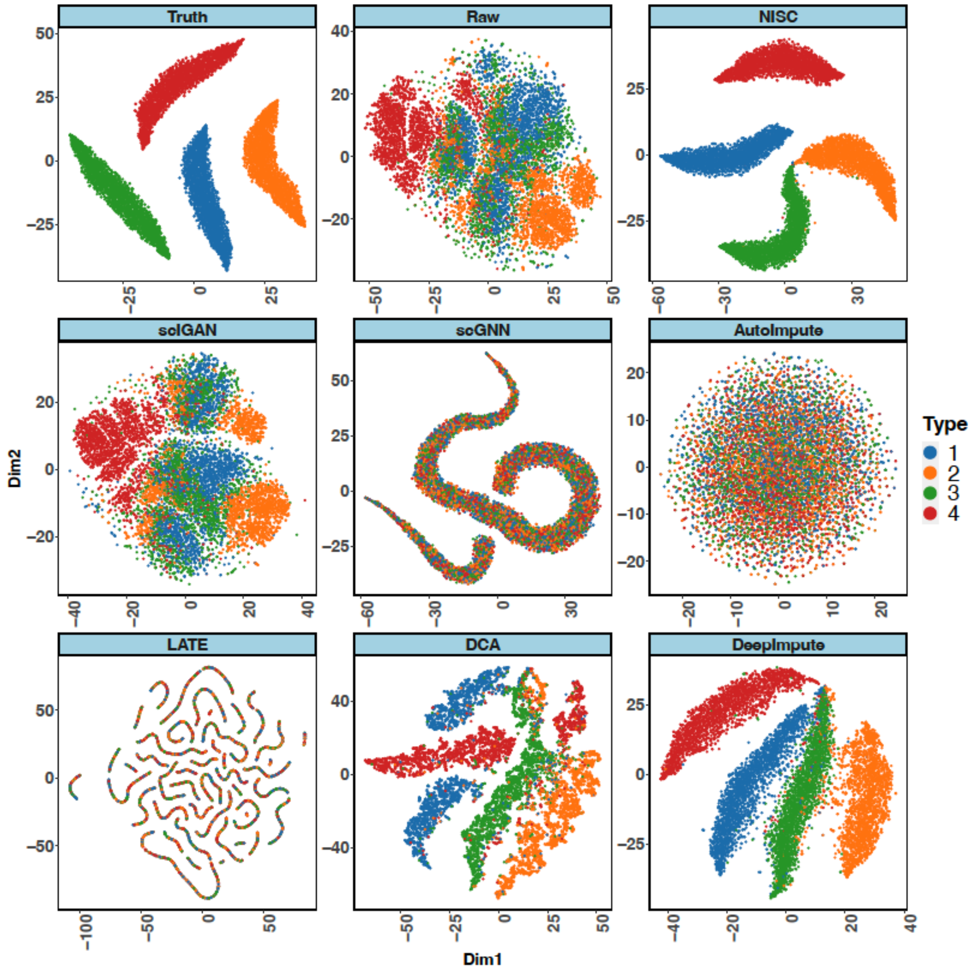

Supplementary Figure 6: t-SNE plot of simulated data of 10,000 cells. The simulated data contain 2,000 genes, four cell types, and 90% sparsity. This figure shows plots of t-SNE components 1 and 2 derived from ground truth, with dropout (i.e., raw), and imputed data using NISC and other deep-learning based imputation methods. Cells are colored by cell types.

A

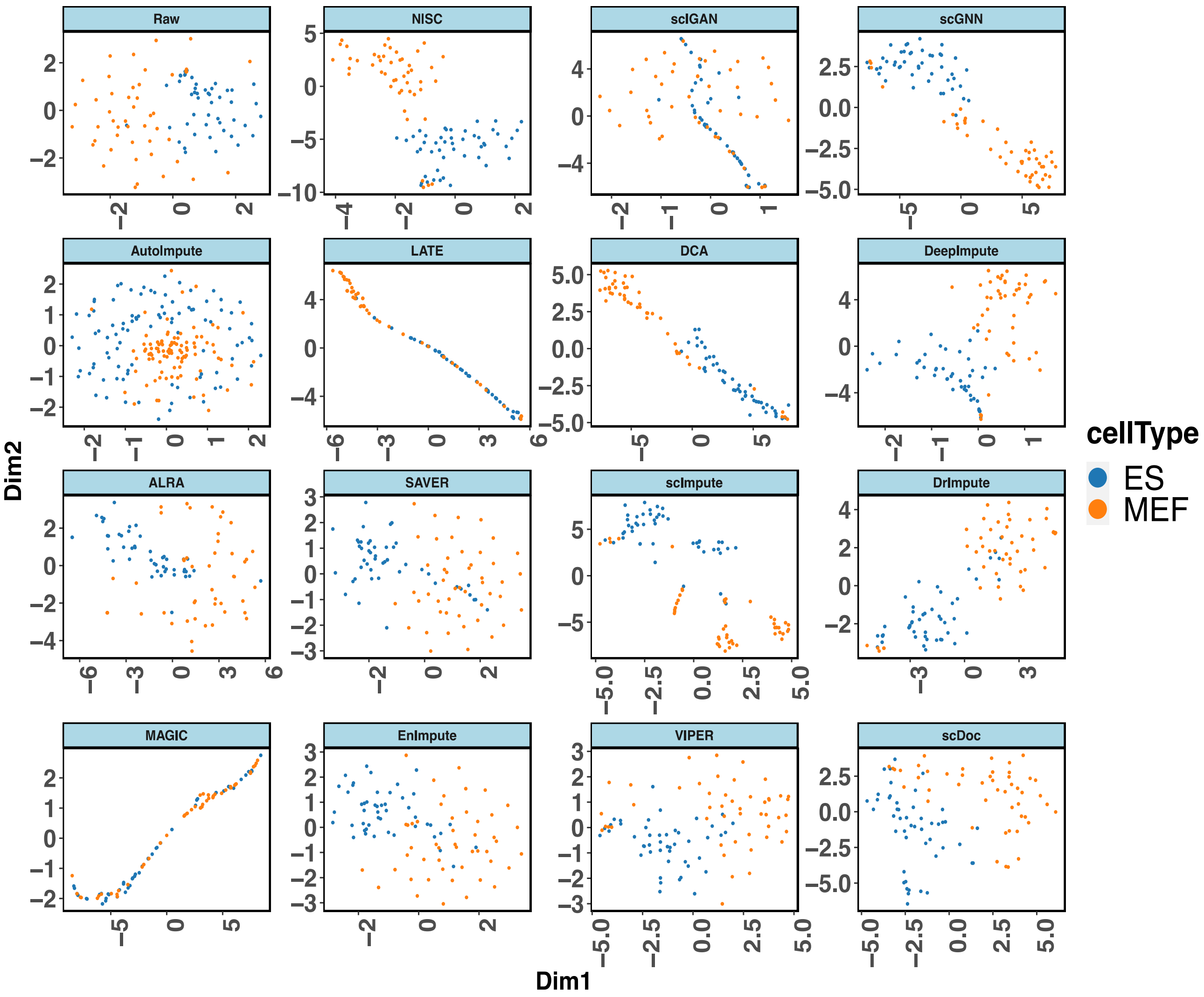
Raw
NISC
scIGAN
scGNN
AutoImpute
LATE
DCA
DeepImpute
ALRA
SAVER
scImpute
DrImpute
MAGIC
EnImpute
VIPER
scDoc
Dim2
Dim1
cellType
ES
MEF

B

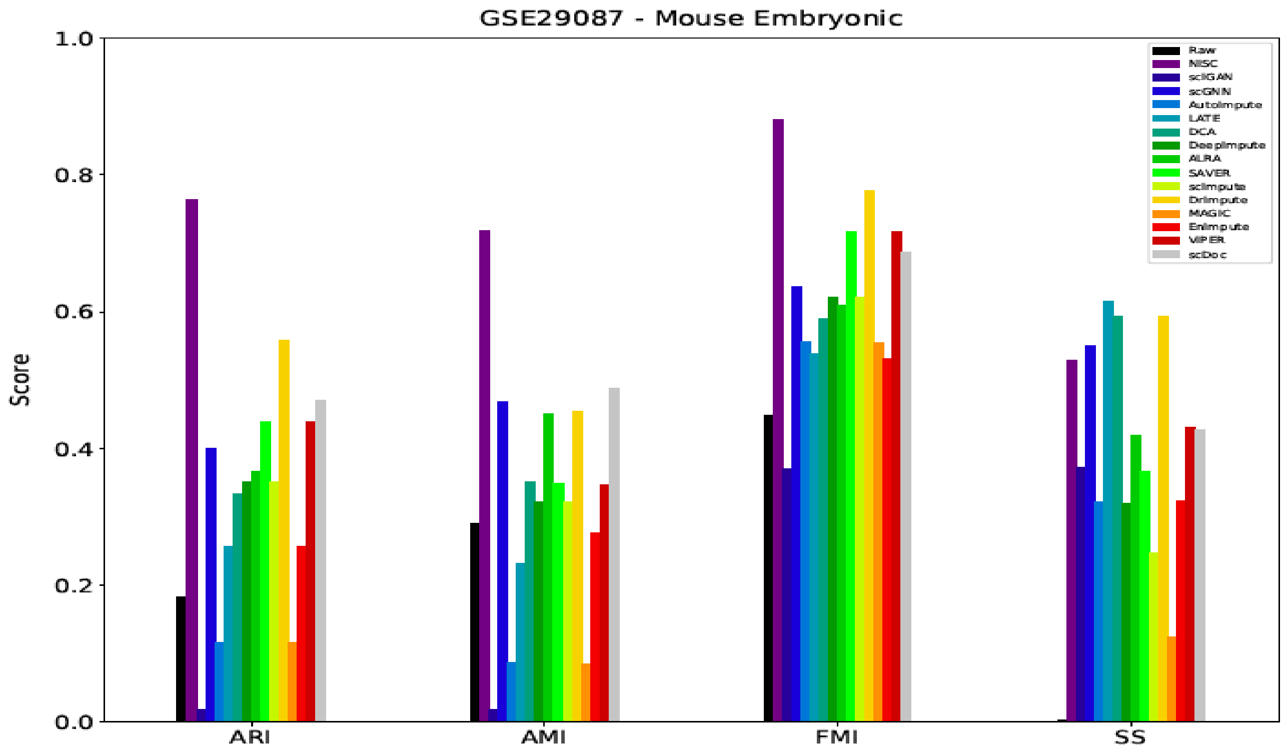

Supplementary Figure 7. NISC recovers the cell types in mouse embryonic cell data (GSE29087), which contain 92 cells and 22,936 genes. The sparsity of the data is 83.04%. (A) t-SNE plots of the original raw data, data imputed by various methods. The details of the imputation methods can be found in the Introduction section in the main text. Cells are colored by cell type which are reported in the original publication (Islam et al., 2011). (B) The plot shows accuracy measurements of Adjusted Rand Index (ARI), Adjusted Mutual Information (AMI), Fowlkes-Mallows Index (FMI), and Silhouette Score (SS) for the raw data and data after imputation.

REFERENCES

Badsha, M., Li, R., Liu, B., Li, Y. I., Xian, M., Banovich, N. E., & Fu, A. Q. (2020). Imputation of single-cell gene expression with an autoencoder neural network. *Quantitative Biolog*y, 8(1), 78-94.

Islam, S., Kjällquist, U., Moliner, A., Zajac, P., Fan, J. B., Lönnerberg, P., & Linnarsson, S. (2011). Characterization of the single-cell transcriptional landscape by highly multiplex RNA-seq. *Genome research*, 21(7), 1160-1167.

Johnson, K. A., & Goody, R. S. (2011). The original Michaelis constant: translation of the 1913 Michaelis–Menten paper. Biochemistry, 50(39), 8264-8269.

Nemec, A. F. L., & Brinkhurst, R. O. (1988). The Fowlkes–Mallows statistic and the comparison of two independently determined dendrograms. *Canadian Journal of Fisheries and Aquatic Sciences*, 45(6), 971-975.

Rousseeuw, P. J. (1987). Silhouettes: A graphical aid to the interpretation and validation of cluster analysis. *Comput. Applied Math., 20*, 53-65.

Romano, S., Bailey, J., Nguyen, V., & Verspoor, K. (2014). Standardized mutual information for clustering comparisons: one step further in adjustment for chance. *In International Conference on Machine Learning* (pp. 1143-1151).

Skinnider, M. A., Squair, J. W., & Foster, L. J. (2019). Evaluating measures of association for single-cell transcriptomics. *Nature methods*, 16(5), 381-386.

Steinley, D. (2004). Properties of the Hubert-Arable Adjusted Rand Index. *Psychological methods, 9*(3), 386-396.
